# School Attendance Registers for the Syndromic Surveillance of Infectious Intestinal Disease in UK Children: Protocol for a Retrospective Analysis

**DOI:** 10.2196/30078

**Published:** 2022-01-20

**Authors:** Anna L Donaldson, John P Harris, Roberto Vivancos, Daniel Hungerford, Ian Hall, Sarah J O'Brien

**Affiliations:** 1 NIHR Health Protection Research Unit in Gastrointestinal Infections University of Liverpool Liverpool United Kingdom; 2 Institute of Population Health University of Liverpool Liverpool United Kingdom; 3 Field Epidemiology Service Public Health England Liverpool United Kingdom; 4 Institute of Infection, Veterinary and Ecological Sciences University of Liverpool Liverpool United Kingdom; 5 Department of Mathematics and School of Health Sciences University of Manchester Manchester United Kingdom

**Keywords:** syndromic surveillance, schools, children, absenteeism, infectious intestinal disease, diarrhea and vomiting, school attendance registers

## Abstract

**Background:**

Infectious intestinal disease (IID) is common, and children are more likely than adults both to have IID and to transmit infection onto others. Before the introduction of the vaccine, rotavirus was the leading cause of severe childhood diarrhea, with norovirus and *Campylobacter* predominate pathogens. Public health surveillance of IID is primarily based on health care data, and as such, illness that is managed within the community will often go undetected. School attendance registers offer a novel data set that has the potential to identify community cases and outbreaks of IID that would otherwise be missed by current health surveillance systems. Although studies have explored the role of school attendance registers in the monitoring of influenza among children, no studies have been identified that consider this approach in the surveillance of IID.

**Objective:**

The aim of this study is to explore the role and utility of school attendance registers in the detection and surveillance of IID in children. The secondary aims are to estimate the burden of IID on school absenteeism and to assess the impact of the rotavirus vaccine on illness absence among school-aged children.

**Methods:**

This study is a retrospective analysis of school attendance registers to investigate whether school absences due to illness can be used to capture seasonal trends and outbreaks of infectious intestinal disease among school-aged children. School absences in Merseyside, United Kingdom will be compared and combined with routine health surveillance data from primary care, laboratories, and telehealth services. These data will be used to model spatial and temporal variations in the incidence of IID and to apportion likely causes to changes in school absenteeism trends. This will be used to assess the potential utility of school attendance data in the surveillance of IID and to estimate the burden of IID absenteeism in schools. It will also inform an analysis of the impact of the rotavirus vaccine on disease within this age group.

**Results:**

This study has received ethical approval from the University of Liverpool Research Ethics Committee (reference number 1819). Use of general practice data has been approved for the evaluation of rotavirus vaccination in Merseyside by NHS Research Ethics Committee, South Central-Berkshire REC Reference 14/SC/1140.

**Conclusions:**

This study is unique in considering whether school attendance registers could be used to enhance the surveillance of IID. Such data have multiple potential applications and could improve the identification of outbreaks within schools, allowing early intervention to reduce transmission both within and outside of school settings. These data have the potential to act as an early warning system, identifying infections circulating within the community before they enter health care settings. School attendance data could also inform the evaluation of vaccination programs, such as rotavirus and, in time, norovirus.

**International Registered Report Identifier (IRRID):**

DERR1-10.2196/30078

## Introduction

Infectious intestinal diseases (IIDs) are common in both high- and low-income countries, causing an estimated 2 billion cases globally each year [1]. Norovirus is the leading cause of IID, with *Campylobacter* the most common bacterial cause [1-3]. In children, rotavirus has been a major cause of severe IID until the licensing of the vaccine in 2006 [4]. The high incidence of IID infection results in significant disease burden and economic costs due to work and school absenteeism, lost earnings, reduced workforce productivity, and increased health care use [5-7]. In the United Kingdom alone, IID has been estimated to result in one million additional general practice consultations each year [6], and norovirus, rotavirus, and *Campylobacter* combined cost the UK economy an estimated £150 million (US $200 million) per annum [5]. Over 80% of total costs are borne by patients, driven by lost income and out-of-pocket expenses [5].

Children are disproportionately affected by IID, with those younger than 5 years accounting for 38% of foodborne cases globally [1]. Children are thought to be important transmitters of IID infection and experience prolonged symptoms and viral shedding, reduced immunity, and higher levels of infectiousness [8-12]. The majority of a child’s close contacts are based at school and home [13,14], and infections, especially viruses, can spread easily through these semienclosed environments [15]. This not only increases the risk of outbreaks within school settings but also provides a pathway through which infections can spread from schools into the wider community [13,16,17]. There is evidence that children may be the first affected by seasonal and pandemic disease [18-21], and hence, enhancing infectious disease surveillance in schools could not only improve the health of children but also provide advanced warning before infections start to circulate in the wider community.

Public health surveillance of IID is primarily based on health care data such as laboratory reports, statutory notifications, hospital admissions, primary care consultations, and calls to remote telehealth services [22,23]. The majority of IID cases, however, will be managed in the community, without involvement from health care services. As a result, current surveillance is likely to be substantially underestimating the impact of IID. Furthermore, there is an inherent bias in the surveillance of IID, as certain groups are more susceptible to complications and therefore more likely to present to health care, such as the very young, the comorbid, and older adults [2,3,24,25]. Laboratory testing policies can also be targeted toward detecting pathogens in these high-risk groups [26], further increasing the surveillance bias. Enhancing the surveillance of IID and improving detection of community cases of disease would provide important information on the epidemiology of these infections. Such data would be of value to support the evaluation of public health interventions, such as rotavirus vaccination and, in time, norovirus vaccination. As vaccinations can alter the epidemiology of infection [27], it is crucial we are able to accurately monitor the long-term impact and effectiveness of these interventions, not only on health care services but also on prevalence in the community.

School attendance registers offer a novel data set that could be used to identify community cases of IID that might not otherwise be detected. School absenteeism data have shown potential in the surveillance of both seasonal and pandemic influenza [28-35], but no studies have been identified that consider their role in monitoring IID. Although mild cases of diarrhea and vomiting will not necessitate contact with health care services, they are likely to still result in an absence from school for the duration of the illness and, in line with public health guidance, an additional 48 hours after symptoms have resolved [36]. This provides a routine data set that has the potential to capture illness from the day of onset.

This study aims to explore the role and utility of school attendance registers in the detection and surveillance of IID in children. The secondary aims are to estimate the burden of IID on school absenteeism and to assess the impact of the rotavirus vaccine on illness absence among school-aged children.

## Methods

### Study Setting

This study will take place in local government areas within Merseyside in the North West of England. Merseyside is a predominately urban, metropolitan county with a population of 1.38 million, over 240,000 of whom are school-aged children [37]. It comprises five local government areas, which range in size from 145,000 residents to over 450,000 residents [37]. For this study, the population of interest is children aged 4-16 years who are registered at a school within Merseyside.

### Study Design

The study will be a retrospective analysis of school absenteeism data to investigate whether school attendance registers can be used to capture seasonal trends and outbreaks of IID among school-aged children. Although these data are routinely collected by local government for school attendance management [38], this is a novel application of this data set. In the United Kingdom, all absences due to illness are given a single code, which distinguishes them from absences due to other causes, including those to attend medical appointments. However, the nature of the illness is not reported. Routine health surveillance data from primary care, laboratories, and the NHS 111 telehealth service will be used to model spatial and temporal variations in the incidence of IID and to apportion likely cause to changes in school absenteeism trends. This will allow an assessment to be made of the potential value and lead time of school absenteeism data in the surveillance of IID and the overall burden of IID on illness absenteeism. The impact of the rotavirus vaccine, which was introduced in the United Kingdom in 2013, will also be explored. As none of the school-aged children included in this study will have received the rotavirus vaccine, this study will capture the impact of vaccinating infants on herd immunity and reducing illness absenteeism among older, unvaccinated children [27].

### Data Sources

School absenteeism data is available at the individual school level. Attendance data for schools providing primary (4-11 years of age) and secondary (11-16 years of age) education, regardless of type of school, will be sought from the local government in Merseyside, with data broken down by school and year group. Total absences and absences due to illness will be requested. Details of the number of children in each school and year group will also be obtained to allow corresponding rates to be calculated.

Laboratory data reported to Public Health England (PHE) North West will be used to obtain organism-specific rates of IID within the different geographical areas. These data are routinely collected and reported to PHE from diagnostic and reference laboratories [39]. PHE also holds data from NHS 111, which is a telehealth service that operates across England [40]. Calls to NHS 111 (and its precursor, NHS Direct) for diarrhea or vomiting will be used to indicate probable cases of IID. NHS 111 and NHS Direct data are held securely by the PHE Real-time Syndromic Surveillance team and can be accessed with permission via PHE.

Primary care consultations for diarrhea or vomiting will be used as another measure of probable cases of IID. These data have recently been collected from clinical commissioning groups and general practices across Merseyside to inform an evaluation of the rotavirus vaccine [41]. Read codes were used to distinguish acute cases of IID from cases linked to chronic conditions or noninfective causes [41]. These data can be accessed from the University of Liverpool in an anonymized format as a secondary data set to further inform the evaluation of the rotavirus vaccine.

To facilitate the spatiotemporal modeling, numbers from each data set will be aggregated to weekly rates to enable a common timescale. The spatial measurement will depend upon the data source; for school absenteeism data, the postcode of the school will be used alongside the catchment area (where appropriate). Primary care consultation data has been mapped to lower super output areas (LSOAs), which represents a geographical area with between 1000 and 3000 residents [42]. Laboratory data contains full postcodes, but to protect the anonymity of patients, these will be reduced to LSOAs before the data is transferred to the research team for analysis. Telehealth data contains only the postcode district of patients [43], which is a larger geographical aggregation than LSOA, ensuring anonymity. Denominator populations will be derived from the Office of National Statistics midyear population estimates [42]. Comparison of derived population estimates will be made with the Health and Safety Laboratories National Population Database [44], and the most suitable denominator population will be used.

To allow the analysis to be conducted at the year-group level, the surveillance data will include details of the patient’s age (year of birth) and their sex. All other personally identifiable information will be removed from the data before it is transferred to the research team. The outcomes of the analysis will be based on aggregated data.

### Study Period

Data will be examined retrospectively from July 2007 to June 2016, capturing 9 IID seasons. Each season is considered to start in calendar week 27 and end in calendar week 26 of the following year.

### Population Sample

This study will focus on three of the five local government areas within Merseyside to reflect the coverage of primary care data collected to inform an evaluation of the rotavirus vaccine [45]. The population sample was estimated using data from the Department for Education, which holds a record of all local government–registered schools [46]. Data were based on the 2017/2018 academic year, limited to schools providing primary and secondary education (ages 4-16 years).

The total number of schools across the three local government areas is 372, consisting of 299 primary schools and 103 secondary schools. Of these schools, 30 deliver both primary and secondary education. The total pupil population across all included schools is 140,164. Assuming that each year one in four pupils are affected by IID [47], in each academic year, we estimate there would be approximately 35,000 cases of IID in schools within the study area. As data will be requested over a 9-year period, the total number of cases across the study period is estimated to be 315,000.

### Case Definitions

The case definitions used within each data set are outlined in Textbox 1.

**Case definitions.**

**School attendance registers**

- Absence with registration code “I” (illness, not medical or dental appointments)

**NHS 111 calls**

- Calls for vomiting
- Calls for diarrhea

**General practice consultations (read codes in parenthesis)**

- Diarrhea and vomiting (19G)
- Diarrhea symptom not otherwise specified (19F6)
- Viral gastroenteritis (A07y0)
- Diarrhea (19F2)
- Gastroenteritis—presumed infectious origin (A0812)
- Diarrhea of presumed infectious origin (A083)
- Infantile viral gastroenteritis (A07y1)
- Infectious gastroenteritis (A0803)
- Enteritis due to rotavirus (A0762)
- Infectious diarrhea (A082)

**Laboratory detections**

- Detection of bacterial, viral, or protozoal infectious intestinal disease organisms in a fecal specimen

### Recruitment and Consent

Recruitment will be conducted at a local government level. Local government will be approached via their public health departments and invited to participate in this study. Consent for use of aggregated school attendance data will be sought from the local government, who carry the legal responsibility for the data and its use. As the data are aggregated and anonymized, consent will not be sought from individual schools or parents.

### Data Analysis

A descriptive analysis will be undertaken of each data set to examine and describe the temporal trends and seasonality of illness absenteeism rates and of confirmed and probable cases of IID. The analysis will be stratified by age to capture varying rates of disease in different year groups. Rotavirus-specific incidence data will be obtained from laboratory reports. The mathematical and statistical analysis will include an organism-specific dynamic transmission model and a mixed effect regression analysis to apportion cause to the variations in absenteeism and to estimate organism-specific incidence rates. The complexity of dynamical models will be decided during the project based on the outputs of the descriptive analysis. Rotavirus modeling will include an interrupted time series analysis to explore changes in school absenteeism rates pre- and postintroduction of the vaccine. This will support an assessment of the impact of vaccination on disease transmission in the community. Other organisms that commonly cause IID in children will also be included in the analysis (eg, norovirus and *Campylobacter*) to test the ability of illness absenteeism data to accurately detect seasonal trends and outbreaks of disease. This will inform an assessment of the suitability of school attendance registers as a potential form of disease surveillance in the community and its role in the long-term monitoring of vaccine-preventable diseases.

## Results

This study received ethical approval from the University of Liverpool Research Ethics Committee (reference number 1819). Use of general practice data has been approved for the evaluation of rotavirus vaccination in Merseyside by the NHS Research Ethics Committee, South Central-Berkshire REC Reference 14/SC/1140. Study findings will be submitted to open access peer-reviewed journals and presented at scientific conferences and meetings, including meetings with stakeholders.

## Discussion

Current surveillance of IID is predominantly based on health care data, and therefore, illness that is managed within the community will often go undetected. This study is unique in considering whether school absenteeism data could be used to enhance the surveillance of IID. The findings could have several important applications. These data could support the improved identification of outbreaks in schools, allowing early intervention to reduce transmission both within and outside of the school setting. As children may be the first affected by seasonal illness, these data have the potential to act as an early warning system, identifying infections circulating within the community before they enter health care settings. Absenteeism data could also be used to inform the evaluation of vaccination programs, such as rotavirus and potentially, in time, norovirus. Similarly, these data could be used to monitor the impact of health improvement programs such as handwashing interventions.

However, there are some limitations that should be considered. The most pertinent is the low specificity of the case definition for illness absenteeism. As a single code is used for all causes of illness absenteeism, these data cannot distinguish between absences caused by IID and absences from other illnesses such as respiratory tract infections. Therefore, the burden of IID on absenteeism cannot be directly measured, and modeling of routine surveillance data is required to apportion likely cause to changes in absenteeism rates. A further consideration in this study is the spatial measure available within each data set; the NHS 111 telehealth service does not collect information below the level of postcode district and hence the statistical modeling, when including this data set, will be restricted to this geographical level. This limits the ability of this analysis to test whether school absenteeism data can detect localized outbreaks of IID within communities. However, this reflects a limitation within our current surveillance systems and one that school attendance data has the potential to rectify. Future work should consider the feasibility of collecting symptom-specific absence information from schools to enhance the specificity of the data and support the syndromic surveillance of a broader range of childhood infectious disease.

## Abbreviations

HPRU: Health Protection Research Unit
IID: infectious intestinal disease
LSOA: lower super output area
NIHR: National Institute for Health Research
PHE: Public Health England
